# A Cardiopulmonary Monitoring System for Patient Transport Within Hospitals Using Mobile Internet of Things Technology: Observational Validation Study

**DOI:** 10.2196/12048

**Published:** 2018-11-14

**Authors:** Jang Ho Lee, Yu Rang Park, Solbi Kweon, Seulgi Kim, Wonjun Ji, Chang-Min Choi

**Affiliations:** 1 Department of Pulmonology and Critical Care Medicine Asan Medical Center University of Ulsan College of Medicine Seoul Republic of Korea; 2 Department of Biomedical System Informatics Yonsei University College of Medicine Seoul Republic of Korea; 3 Department of Oncology Asan Medical Center University of Ulsan College of Medicine Seoul Republic of Korea

**Keywords:** wearable device, patient safety, intrahospital transport, oxygen saturation, heart rate, mobile application, real-time monitoring

## Abstract

**Background:**

During intrahospital transport, adverse events are inevitable. Real-time monitoring can be helpful for preventing these events during intrahospital transport.

**Objective:**

We attempted to determine the viability of risk signal detection using wearable devices and mobile apps during intrahospital transport. An alarm was sent to clinicians in the event of oxygen saturation below 90%, heart rate above 140 or below 60 beats per minute (bpm), and network errors. We validated the reliability of the risk signal transmitted over the network.

**Methods:**

We used two wearable devices to monitor oxygen saturation and heart rate for 23 patients during intrahospital transport for diagnostic workup or rehabilitation. To determine the agreement between the devices, records collected every 4 seconds were matched and imputation was performed if no records were collected at the same time by both devices. We used intraclass correlation coefficients (ICC) to evaluate the relationships between the two devices.

**Results:**

Data for 21 patients were delivered to the cloud over LTE, and data for two patients were delivered over Wi-Fi. Monitoring devices were used for 20 patients during intrahospital transport for diagnostic work up and for three patients during rehabilitation. Three patients using supplemental oxygen before the study were included. In our study, the ICC for the heart rate between the two devices was 0.940 (95% CI 0.939-0.942) and that of oxygen saturation was 0.719 (95% CI 0.711-0.727). Systemic error analyzed with Bland-Altman analysis was 0.428 for heart rate and –1.404 for oxygen saturation. During the study, 14 patients had 20 risk signals: nine signals for eight patients with less than 90% oxygen saturation, four for four patients with a heart rate of 60 bpm or less, and seven for five patients due to network error.

**Conclusions:**

We developed a system that notifies the health care provider of the risk level of a patient during transportation using a wearable device and a mobile app. Although there were some problems such as missing values and network errors, this paper is meaningful in that the previously mentioned risk detection system was validated with actual patients.

## Introduction

As medicine continues to progress, patient safety has become more important. Intrahospital transport (IHT) is necessary during clinical practice. Pulmonary rehabilitation is regarded as one of the most important interventions for chronic pulmonary disease patients [1-4]. Nevertheless, adverse events during IHT and rehabilitation are inevitable. One study reported that 1.7% of critically ill patients suffered adverse events during IHT, defined as life-threatening events. Another study reported that adverse events, from equipment problems to life-threatening situations, occurred in 79.8% of patients during IHT. [2,5-7]. Although it is desirable for clinicians to accompany and observe their patients during IHT to reduce these events, this is impossible in the real world due to medical resource limitations.

Recent changes in the mobile and internet environment have an effect on the medical field [8-11]. Due to progress in high-speed data transmission capabilities and the ability to wirelessly connect to external devices, several telemonitoring techniques have been developed in various fields [12-14]. These techniques offer immediate information about patients to clinicians and facilitate adequate management of patients.

There are several telemonitoring solutions for oxygen saturation and heart rate, but most of them were developed for long term rather than immediate management [15,16]. The Prince 100-H wrist oximeter and SpO_2_ monitor version 0.23 were developed for simultaneous supervision of oxygen saturation and heart rate during patient transport or rehabilitation and can notify clinicians of adverse events.

In this study, we aimed to answer two questions: (1) Are wearable devices and mobile applications suitable for recognizing risk during transit? and (2) Is transmission of the risk signal over the network reliable? To address these questions, we collected biometrics data during the transport of respiratory medicine inpatients using wearable devices and a mobile app. We developed a risk detection algorithm to analyze the collected data and notified the health care provider when necessary.

## Methods

### Study Design

We performed a single-center study at Asan Medical Center in South Korea. Patient screening was based on admission to the pulmonology ward between May 16, 2018 and May 31, 2018. Exclusion criteria were as follows: (1) patients in the acute phase based on the clinicians’ judgment, and (2) patients not transported for work up or rehabilitation.

**Figure 1:**
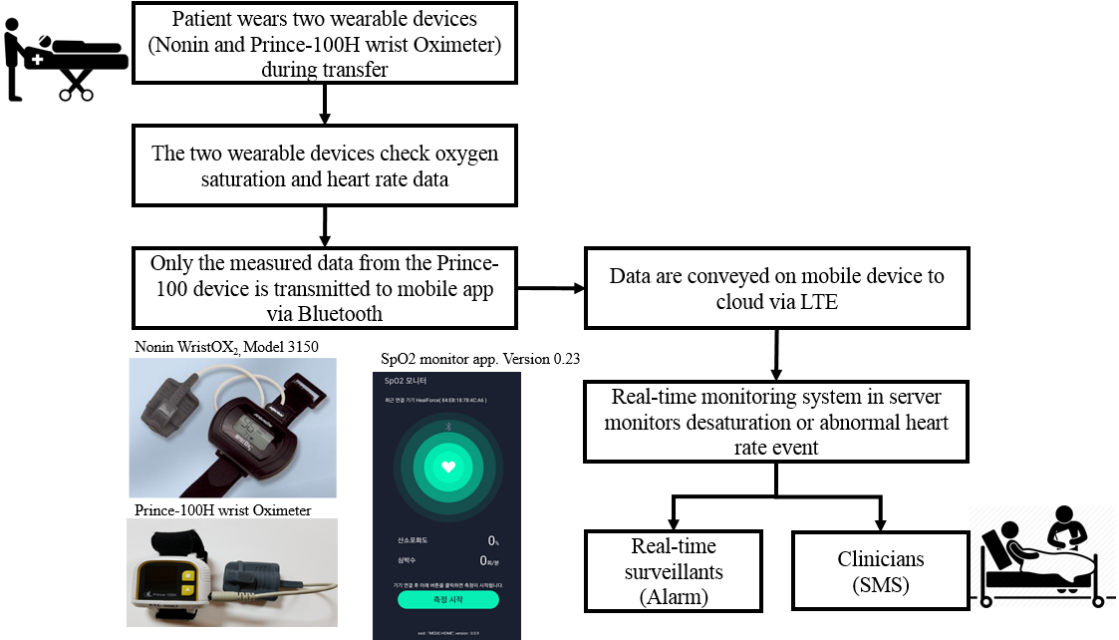
Data flow for risk signal detection system during patient transfer through wearable device and a mobile app.

Oxygen saturation and pulsation were measured in real time using portable oxygen saturation measurement equipment for patients with risk factors that reduce oxygen saturation and were measured in the hospital for diagnostic purposes or rehabilitation. The measured data were collected in real time on a connected mobile phone and transmitted to a real-time monitoring system via the Internet of Things (IoT). The transmitted data were checked in real time on monitoring equipment on the ward where the patient was hospitalized by clinicians on the study team. When there was a risk, an alarm system was activated, allowing the staff to immediately (within 1 minute) identify and respond to the danger. Using a wearable sensing device and mobile phone app, we collected data from 23 patients at Asan Medical Center in Seoul and analyzed the risk factors during patient transport (Figure 1).

### Device and Mobile App

When patients needed to be transported for rehabilitation or study, we employed the Prince-100H wrist oximeter and the Nonin for monitoring. Both devices check oxygen saturation and heart rate in real time. The Prince-100H wrist oximeter generates SpO_2_ and pulse data at 1 record per second, while the Nonin, which is approved by the US Food and Drug Administration (FDA), generates one record every 4 seconds. Only measured data from the Prince-100H wrist oximeter were transmitted to the mobile app, SpO_2_ monitor version 0.23, via Bluetooth, and delivered to the cloud over the network. When the monitoring system detected desaturation or abnormal heart rate events, a notification was transmitted to real-time surveillants by displaying an alarm on the monitor and to clinicians by short message service (SMS) text messaging. We selected SMS text messaging as tools for notification to clinicians because our judgment was that SMS text messaging was relatively less affected by the mobile environment. Notification of a risk signal occurred only in the following four cases: (1) oxygen saturation less than 90%, (2) heart rate greater than 140 beats per minute (bpm), (3) heart rate less than 60 bpm, and (4) network error.

The device and mobile phone app were used for IHT during the hospitalization of 23 patients. We investigated the possible risk factors and disease severity during transport to determine the efficiency of the monitoring and transmission system. Furthermore, the risk factors during transport were identified through comparative analysis of the patients with and without the hazards identified during transport.

This study was approved by the institutional review board of the Asan Medical Center (IRB no. 2018-0480). We obtained informed consent from all study participants.

### Data Description

In this study, we collected three types of data: (1) clinical information of patients collected on admission to Asan Medical Center, (2) the data collected during the clinical trial through the case report form for participants, and (3) the patient’s real-time pulse and oxygen saturation by pulse oximetry (SpO_2_) values were measured using two wearable devices (Nonin and Prince-100H wrist oximeters). The clinical information for the patients included age, sex, body mass index, smoking history, oxygen use before trial, and underlying disease (including diabetes mellitus, hypertension, tuberculosis history, respiratory disease, arrhythmia, lung cancer, and other malignancies). The data collected during the clinical trial included the reason for study device application, vital signs before the trial, and the results of a pulmonary function test given to available patients. We only employed the study device for monitoring during IHT or rehabilitation.

### Data Analysis

**Figure 2:**
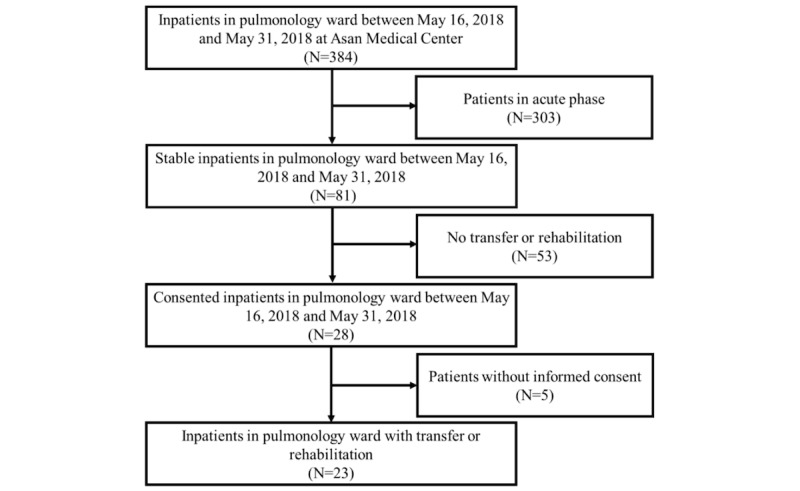
Recruitment for patient safety study related to patient transfer within hospital.

Figure 2 shows the patient selection flowchart for the study. Among 384 patients admitted to the pulmonology ward between May 16, 2018 and May 31, 2018, we excluded the following patients: (1) 303 patients in the acute phase, (2) 53 patients without in-hospital transfer or a rehabilitation schedule, and (3) five patients without informed consent. Twenty-three patients were finally selected for this study.

To analyze the interdevice agreement rate, the Nonin and Prince-100H data collected every 4 seconds, which is the data generation criterion for the Nonin. If the Prince-100H did not have a value at exactly the same time, imputation was performed at an average of ±1 second. Based on matching data, the Pearson correlation coefficient (*r*) between the different wearable devices was determined with linear regression. The intraclass correlation coefficients (ICCs) were used to assess the relationships between the two wearable devices [17,18]. Point estimates of the ICCs were interpreted as follows: excellent (0.75-1), modest (0.4-0.74), or poor (0-0.39). The Bland-Altman method was also used to measure the agreement of the pulse and SpO_2_ values for the two wearable devices. The Student *t* test was used to compare differences in the groups for abnormal and unmatched signals. All reported *P* values were two-sided, and *P* values less than .05 were considered significant. All statistical analyses were performed using R version 3.5.0. We expressed the categorical variables as numbers with the proportion of subjects and continuous variables presented as means with standard deviations.

## Results

### Overall Characteristics

Twenty-three patients consented to inclusion in this study. The baseline characteristics of the enrolled patients are summarized in Table 1. Categorical variables are expressed as a number with the proportion of participants. Continuous variables are expressed as means with standard deviations. Pulmonary function test results were extracted from 18 patients, because five did not take a pulmonary function test before the study. The mean age was 64.4 (SD 11.1) years, and 12 men were included. The mean body mass index was 23.7 (SD 2.4) kg/m^2^. Seven patients had underlying respiratory disease and three of them used an oxygen supply before the study. Nineteen patients had malignancies, and none had underlying arrhythmia. Twenty patients used the study device and app during IHT for diagnostic purpose, and the other three patients used the device for rehabilitation. In the baseline pulmonary function test of 18 patients, the mean forced expiratory volume in first second of expiration (FEV_1_) was 2.1 (SD 0.7) L, mean forced vital capacity (FVC) was 3.1 (SD 0.9) L, mean FEV_1_/FVC was 70.4% (SD 11.0%), and mean diffusing capacity for carbon monoxide (DLCO) was 67.1% (SD 20.3%).

### Difference in Data Transmission by Network Type

Of the 23 patients, only two used the Wi-Fi network and 21 used the LTE network to transmit data generated from the Prince-100H device to the cloud (Multimedia Appendix 1). The total measurement time of this study was 875.1 minutes (mean 38.0, SD 29.4 minutes). During this time, the Nonin produced 14,161 records and the Prince-100 produced 49,282 records. Since the Prince-100H measures SpO_2_ and pulses data in 1 second units, this was matched with one record every 4 seconds from the Nonin. The mean value for 109.34 records before the imputation was “not available” and the mean value of 24.21 records after imputation was the not available value. The remaining mean not available ratio was 3.79%. Patients with the highest not available ratios were in the following order: P05 (28.02%), P07 (14.56%), and P03 (12.74%). Two patients with Wi-Fi transmission had higher not available ratios before imputation and their not available ratio values ​​after imputation were lower than those for LTE patients.

### Correlation Analysis of Two Wearable Devices

**Figure 3:**
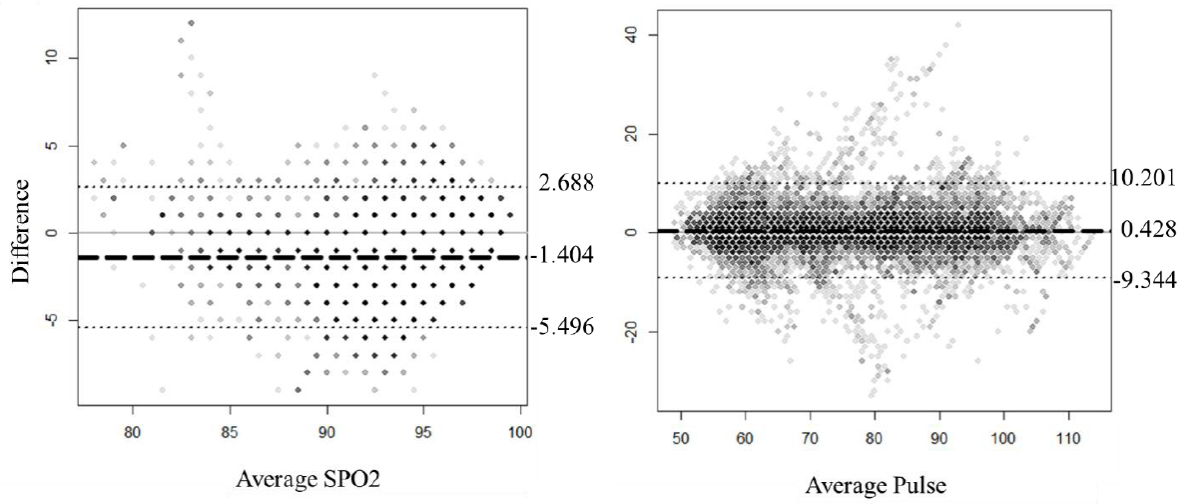
Bland-Altman plots representing comparisons between the Nonin and Prince-100H devices for SpO_2_ (left) and pulse (right). The dashed line represents the mean difference between the devices, with the upper and lower lines (dotted lines) representing the limits of agreement (±2SD).

The SpO_2_ and pulse variables measured by the Prince-100H and Nonin are given in Table 2. The pulse ICC between the two devices was 0.940 (95% CI 0.939-0.942), which indicated “excellent” agreement, and the SpO_2_ ICC was 0.719 (95% CI 0.711-0.727), which indicated “good” agreement (Table 2). In addition, Bland-Altman analysis of the pulse revealed that the systematic error was low at 0.428 compared to –1.404 for SpO_2_ (Figure 3). The 95% limit of agreement was in the –9.344 to 10.201 range for the pulse and –5.496 to 2.688 for the SpO_2_.

**Table 1:** Basic characteristics of patients (N=23).

Variable		Total
Age (years), mean (SD)		64.4 (11.1)
Male, n (%)		12 (52.2)
Body mass index (kg/m^2^), mean (SD)		23.7 (2.4)
Smoking history, n (%)		13 (56.5)
Oxygen use before trial, n (%)		3 (13.0)
**Vital sign before trial, mean (SD)**		
	Systolic blood pressure (mm Hg)	117.1 (13.5)
	Diastolic blood pressure (mm Hg)	73.0 (7.3)
	Heart rate	75.0 (11.3)
	Respiratory rate	18.3 (1.4)
**Underlying disease, n (%)**		
	Diabetes mellitus	5 (21.7)
	Hypertension	9 (39.1)
	Tuberculosis history	3 (13.0)
	Respiratory disease	7 (30.4)
	Arrhythmia	0 (0.0)
	Lung cancer	17 (73.9)
	Other malignancy	2 (8.7)
**Reason for study device application, n (%)**		
	Intrahospital transport	20 (87.0)
	Rehabilitation	3 (13.0)
**Pulmonary function test (N=18), mean (SD)**		
	FVC^a^ (L)	3.1 (0.9)
	FEV_1_^b^ (L)	2.1 (0.7)
	FEV_1_/FVC (%)	70.4 (11.0)
	DLCO^c^ (%)	67.1 (20.3)

^a^FVC: forced vital capacity.

^b^FEV_1_: forced expiratory volume in 1 sec.

^c^DLCO: diffusing capacity for carbon monoxide.

**Table 2:** Characteristics of the patients with abnormal signals (“yes”) versus those without (“no”) during transport (N=23).

Variables and categories		Yes (n=14)	No (n=9)	*P* value^a^
Age (years), mean (SD)		62.07 (12.91)	68 (6.75)	.17
**Sex, n (%)**				.40
	Male	6 (43.86)	6 (66.67)	
	Female	8 (57.14)	3 (33.33)	
Weight, mean (SD)		62.71 (11.76)	61.11 (7.47)	.69
Body mass index, mean (SD)		24.01 (2.71)	23.33 (1.91)	.49
Oxygen use before study n (%)		3 (21.43)	0 (0)	.25
Study device application: rehabilitation n (%)		3 (21.43)	0 (0)	.25
**Smoking n (%)**				.22
	Nonsmoker	8 (57.14)	2 (22.22)	
	Ex-smoker	3 (21.43)	5 (55.56)	
	Current-smoker	3 (21.43)	2 (22.22)	
**Underlying disease n (%)**				
	Diabetes mellitus	3 (21.41)	2 (22.22)	>.99
	Hypertension	4 (28.57)	5 (55.56)	.38
	History of tuberculosis	0 (0)	3 (33.33)	.05
	Pulmonary disease	4 (28.57)	3 (33.33)	>.99
	Arrhythmia	0 (0)	0 (0)	>.99
	Lung cancer	10 (71.43)	7 (77.78)	>.99
	Other malignancy	1 (7.14)	1 (11.11)	>.99
**Pulmonary function test, mean (SD)**				
	FVC^b^	2.99 (0.97)	3.22 (0.73)	.57
	FEV_1_^c^	2.13 (0.77)	2.17 (0.48)	.88
	FEV_1_/FVC	0.72 (0.13)	0.68 (0.04)	.35
	DLCO^d^	63.42 (20.32)	74.33 (19.92)	.30
**Vital sign before trial, mean (SD)**				
	Systolic blood pressure (mmHg)	115.79 (11.81)	119.11 (16.22)	.60
	Diastolic blood pressure (mmHg)	73.36 (4.97)	72.44 (10.33)	.81
	Heart rate	73.07 (11.09)	78.11 (11.56)	.31
	Respiratory rate	17.86 (1.46)	18.89 (1.05)	.06
	Temperature	36.60 (0.27)	36.64 (0.44)	.79

^a^Student *t* test for continuous variables and Fisher exact test for categorical variables.

^b^FVC: forced vital capacity; FEV_1_: forced expiratory volume in 1 sec; DLCO: diffusing capacity for carbon monoxide.

^c^FEV_1_: forced expiratory volume in 1 sec.

^d^DLCO: diffusing capacity for carbon monoxide.

### Risk Signal Detection Using Prince-100H Wrist Oximeter and SpO _2_ Monitor

Among the 23 patients, 14 had 20 risk signals during transport within the hospital, and none of the patients had a heart rate above 140. The risk signals occurred nine times for eight patients with less than 90% saturation, four times for four patients with heart rates below 60 bpm, and seven times for five patients due to network errors. Except for the risk signals for network error, we compared the characteristics of the patients with and without risk signals (Table 2). Although most variables were not statistically significant between the alarm and nonalarm groups, three patients in the alarm group used oxygen supplement devices before monitoring device use compared to none in the nonalarm group. Patients in the alarm group had lower pulmonary function test results than those in the nonalarm group, especially the DLCO value. After notification of risk signal, clinicians visited patients at risk and properly managed them. For example, for patients with hypoxemia, clinicians applied oxygen therapy until the patients stabilized without hypoxemia.

## Discussion

In this study, we confirmed that a wearable device and mobile app can detect risk signals effectively during the transport or rehabilitation of a patient within the hospital. In addition, a real-time risk signal was sent to the health care provider in a message to ensure patient safety. To our knowledge, this is the first study involving simultaneous monitoring of oxygen saturation and heart rate in patients during transport or rehabilitation. In our study, the Prince-100H wrist oximeter and a mobile app, SpO_2_ monitor, showed comparable results for oxygen saturation and heart rate with the Nonin, which is approved by the FDA for patient monitoring. This means that oxygen saturation and heart rate monitoring using a wearable device and mobile app is stable and reliable for patients.

Telemetry monitoring is a well-known helpful technique for real-time monitoring. The American Heart Association recommends the use of real-time electrocardiographic monitoring for patients with underlying cardiac disease or high-risk patients [19]. After introducing telemetry monitoring, cardiologists can early detect abnormal cardiac rhythm 24 hours per day in real time. Practical applications of real-time electrocardiographic monitoring have led to the expectation that real-time monitoring systems can be utilized in more situations with various parameters. Sala and colleagues [20] suggested that monitoring oxygen saturation and heart rate during rehabilitation after cardiac surgery can be helpful to ensure patient safety. However, the limitation of this suggestion is that physiotherapists have to directly monitor the oxygen saturation and heart rate of patients. To address these problems, wearable devices connected to a network and algorithms to detect risk signals are needed. In our study, we developed a real-time risk monitoring system that included cloud transmission, a mobile app, and a wearable device connected to the network through Wi-Fi or LTE. Because clinicians received the risk signal from the device through a Wi-Fi or LTE network in real time, they did not need to stand by the patients. In addition, they could monitor several patients simultaneously.

In this study, we selected patients admitted to the pulmonology ward, because patients with pulmonary disease have a relatively high risk of desaturation. Most of the patients enrolled after applying the exclusion criteria were patients admitted for diagnostic workup for lung cancer. Two patients were admitted for acute exacerbation of chronic obstructive pulmonary disease and another was admitted for an acute phase of interstitial lung disease.

In our study, there was no significant intergroup differences in the baseline characteristics between the alarm and nonalarm groups. However, all patients who used oxygen supplement devices before the study and one patient who used the monitoring device for rehabilitation were included in the alarm group. In addition, patients in the alarm group had poor pulmonary function test results, especially DLCO. This observation was reasonable because poor pulmonary function, demand for oxygen supply, or rehabilitation imply a higher risk of desaturation or elevated heart rate than normal. Although the nonalarm group had more patients with a history of tuberculosis with statistical significance, this did not appear to have an effect on alarm events. Tuberculosis can cause bronchiectasis or airflow obstruction [21,22], which can lead to chronic airflow obstruction. However, all patients with obstructive patterns had only mild pulmonary dysfunction and there was no difference between the two groups in FEV_1_/FVC, which represents the degree of airflow obstruction [23]. These results indicate that the device sent a risk signal to clinicians regardless of the patient’s history.

We initially used Wi-Fi to transfer the data from the mobile app to the cloud. Because there were a lot of “not applicable” records, we used only LTE for the transfer after the second patient. For the two patients whose data were transmitted using Wi-Fi, most of the values were recorded as not applicable during the imputation process. We concluded that transmission of the measured data to the cloud was delayed because of network traffic. In addition to the two Wi-Fi patients, three LTE patients had exceptionally high not applicable ratios: P05 (28.02%), P07 (14.56%), and P03 (12.74%). Most of the not applicable signals occurred in the elevator or corridor during transport. There is a possibility that a poor mobile app signal delayed data delivery to the cloud. In this situation, we recorded the signal as not applicable and used an imputation value for matching and analysis.

During the trial, there were 20 risk signals for 14 patients. Except for network errors, risk signals for oxygen saturation below 90% were the most common. After clinicians received the risk signals, they applied oxygen supplements and confirmed improved oxygen saturation. In our study, there was no significant difference between risk signal detected and not detected patients. Nevertheless, if we selected patients at high risk and applied the study device to them, it may be more adequate. Patients who were expected to have desaturation events due to the use of an oxygen supplement device before monitoring and IHT for rehabilitation were in the alarm group. In addition, the results of the pulmonary function test for the nonalarm group were better than those for the alarm group. Risk signals for a heart rate of 60 bpm or less occurred for four patients. Among them, three patients had heart rates of 60 bpm or less measured before the study. This suggests that the target heart rate should be personalized before device application. One patient presented decreased heart rate after device application. Clinicians visited this patient and concluded that the decrease was a side effect of pethidine, which had been injected before the diagnostic procedure. The patient recovered after adequate hydration. This is a representative example of the usefulness of real-time monitoring. If clinicians are able to detect early signs of risk in patients, it can reduce the task of managing critically ill patients. Because this study was a feasibility study, we defined risk signal based on nonindividualized criteria rather than real-world situations. For this reason, we believe that there were relatively frequent risk signals. For applying the study device to a real-world setting, we plan further study based on individualized criteria.

This study had several limitations. First, this study was a single-center study with a small number of patients. We wanted to determine the feasibility of real-time monitoring with wearable devices and mobile apps. For this reason, we conducted the study with a small number of patients in a single center. Owing to favorable results in this study, our research team is planning a multicenter study with more patients. Secondly, we measured only oxygen saturation and heart rate for real-time monitoring. We selected these parameters because oxygen saturation and heart rate can be measured simply with only a pulse oximeter and are some of the earliest risk indicators for patients. Third, the patients were not transported through the same route. However, we performed this study to validate wearable devices and mobile apps in a variety of environments. Therefore, we tried to apply them without restrictions. Consequently, we were able to identify issues such as increased not applicable ratio in the elevator or corridor in transit to the room for bronchoscopy. Finally, we applied the study device to only two patients through hospital Wi-Fi, and the others through LTE. In this case, there was a possibility of security problem regarding patients’ medical information. Nevertheless, we changed to LTE due to instability of Wi-Fi and planned to further study for Wi-Fi performance in real-time monitoring.

Despite these limitations, this study demonstrated favorable validation of telemonitoring application with LTE and Wi-Fi during patient transport. During IHT and rehabilitation, utilization of real-time monitoring can help clinicians with early risk detection or decisions such as prescribing oxygen supplements. Further study is needed for generalization to critically ill patients and other applications.

New techniques have been developed to ensure patient safety during transit. In this study, we constructed a system that notifies the health care provider by detecting the risk signal for the patient during transport based on a wearable device and a mobile app. Although there were some problems such as missing values and network errors, this paper is meaningful because the previously mentioned risk detection system was verified on actual patients.

## Appendix

Multimedia Appendix 1 Number of raw, matched, and imputation records from two wearable devices for twenty-three patients.

## Abbreviation

bpm: beats per minute
DLCO: diffusing capacity for carbon monoxide
FEV _1_: forced expiratory volume in 1 sec
FVC: forced vital capacity
ICC: intraclass correlation coefficient
IHT: intrahospital transport
IoT: Internet of Things
SMS: short message service
SpO _2_: oxygen saturation by pulse oximetry

## References

[1] Bolton CE, Bevan-Smith EF, Blakey JD, Crowe P, Elkin SL, Garrod R, Greening NJ, Heslop K, Hull JH, Man WD, Morgan MD, Proud D, Roberts CM, Sewell L, Singh SJ, Walker PP, Walmsley S (2013). British Thoracic Society guideline on pulmonary rehabilitation in
adults. Thorax.

[2] Jia L, Wang H, Gao Y, Liu H, Yu K (2016). High incidence of adverse events during intra-hospital transport of
critically ill patients and new related risk factors: a prospective, multicenter study
in China. Crit Care.

[3] Casaburi R, ZuWallack R (2009). Pulmonary rehabilitation for management of chronic obstructive pulmonary
disease. N Engl J Med.

[4] Lee Y, Jung M, Shin GW, Bahn S, Park T, Cho I, Lee J (2018). Safety and usability guidelines of clinical information systems integrating
clinical workflow: a systematic review. Healthc Inform Res.

[5] Kue R, Brown P, Ness C, Scheulen J (2011). Adverse clinical events during intrahospital transport by a specialized
team: a preliminary report. Am J Crit Care.

[6] Lahner D, Nikolic A, Marhofer P, Koinig H, Germann P, Weinstabl C, Krenn CG (2007). Incidence of complications in intrahospital transport of critically ill
patients--experience in an Austrian university hospital. Wien Klin Wochenschr.

[7] Parmentier-Decrucq E, Poissy J, Favory R, Nseir S, Onimus T, Guerry M, Durocher A, Mathieu D (2013). Adverse events during intrahospital transport of critically ill patients:
incidence and risk factors. Ann Intensive Care.

[8] Kim J, Kam HJ, Park YR, Yoo S, Oh JS, Kim Y, Lee J (2018). Enchanted life space: adding value to smart health by integrating human
desires. Healthc Inform Res.

[9] Il-Young JH, Lee E, Jung HW, Park H, Cheon S, Lee YS, Park YR (2018). Wearable device-based walking programs in rural older adults can improve
physical activity and health outcome: a feasibility study. JMIR Mhealth Uhealth.

[10] Loveday A, Sherar LB, Sanders JP, Sanderson PW, Esliger DW (2015). Technologies that assess the location of physical activity and sedentary
behavior: a systematic review. J Med Internet Res.

[11] Chai PR, Zhang H, Baugh CW, Jambaulikar GD, McCabe JC, Gorman JM, Boyer EW, Landman A (2018). Internet of Things buttons for real-time notifications in hospital
operations: proposal for hospital implementation. J Med Internet Res.

[12] Paré G, Jaana M, Sicotte C (2007). Systematic review of home telemonitoring for chronic diseases: the evidence
base. J Am Med Inform Assoc.

[13] Vandenberk T, Stans J, Mortelmans C, Van Haelst R, Van Schelvergem G, Pelckmans C, Smeets CJ, Lanssens D, De Cannière H, Storms V, Thijs IM, Vaes B, Vandervoort PM (2017). Clinical validation of heart rate apps: mixed-methods evaluation
study. JMIR Mhealth Uhealth.

[14] Noah B, Keller MS, Mosadeghi S, Stein L, Johl S, Delshad S, Tashjian VC, Lew D, Kwan JT, Jusufagic A, Spiegel BM (2018). Impact of remote patient monitoring on clinical outcomes: an updated
meta-analysis of randomized controlled trials. npj Digital Med.

[15] Buekers J, De Boever P, Vaes AW, Aerts J, Wouters EF, Spruit MA, Theunis J (2018). Oxygen saturation measurements in telemonitoring of patients with COPD: a
systematic review. Expert Rev Respir Med.

[16] Vitacca M (2016). Telemonitoring in patients with chronic respiratory insufficiency:
expectations deluded?. Thorax.

[17] Haas M (1991). Statistical methodology for reliability studies. J Manipulative Physiol Ther.

[18] Shrout PE, Fleiss JL (1979). Intraclass correlations: uses in assessing rater
reliability. Psychol Bull.

[19] Sandau KE, Funk M, Auerbach A, Barsness GW, Blum K, Cvach M, Lampert R, May JL, McDaniel GM, Perez MV, Sendelbach S, Sommargren CE, Wang PJ, American Heart Association Council on Cardiovascular and Stroke Nursing; Council
on Clinical Cardiology;Council on Cardiovascular Disease in the Young (2017). Update to practice standards for electrocardiographic monitoring in
hospital settings: a scientific statement from the American Heart
Association. Circulation.

[20] Sala V, Petrucci L, Monteleone S, Dall'Angelo A, Miracca S, Conte T, Carlisi E, Ricotti S, D'Armini AM, Dalla TE (2016). Oxygen saturation and heart rate monitoring during a single session of
early rehabilitation after cardiac surgery. Eur J Phys Rehabil Med.

[21] Chakrabarti B, Calverley PM, Davies PD (2007). Tuberculosis and its incidence, special nature, and relationship with
chronic obstructive pulmonary disease. Int J Chron Obstruct Pulmon Dis.

[22] Polverino E, Goeminne PC, McDonnell MJ, Aliberti S, Marshall SE, Loebinger MR, Murris M, Cantón R, Torres A, Dimakou K, De SA, Hill AT, Haworth CS, Vendrell M, Ringshausen FC, Subotic D, Wilson R, Vilaró J, Stallberg B, Welte T, Rohde G, Blasi F, Elborn S, Almagro M, Timothy A, Ruddy T, Tonia T, Rigau D, Chalmers JD (2017). European Respiratory Society guidelines for the management of adult
bronchiectasis. Eur Respir J.

[23] Pellegrino R, Viegi G, Brusasco V, Crapo RO, Burgos F, Casaburi R, Coates A, van der Grinten CP, Gustafsson P, Hankinson J, Jensen R, Johnson DC, MacIntyre N, McKay R, Miller MR, Navajas D, Pedersen OF, Wanger J (2005). Interpretative strategies for lung function tests. Eur Respir J.

