# Correction: A Cardiopulmonary Monitoring System for Patient Transport Within Hospitals Using Mobile Internet of Things Technology: Observational Validation Study

**DOI:** 10.2196/12864

**Published:** 2018-12-21

**Authors:** Jang Ho Lee, Yu Rang Park, Solbi Kweon, Seulgi Kim, Wonjun Ji, Chang-Min Choi

**Affiliations:** 1 Department of Pulmonology and Critical Care Medicine Asan Medical Center University of Ulsan College of Medicine Seoul Republic of Korea; 2 Department of Biomedical System Informatics Yonsei University College of Medicine Seoul Republic of Korea; 3 Department of Oncology Asan Medical Center University of Ulsan College of Medicine Seoul Republic of Korea

The authors of “A Cardiopulmonary Monitoring System for Patient Transport Within Hospitals Using Mobile Internet of Things Technology: Observational Validation Study” (JMIR Mhealth Uhealth 2018;6(11):e12048) wish to add their grant number to the Acknowledgments section.

Thus, the sentence “This study was supported by a grant from Korea Health Industry Development Institute” has been changed to “This study was supported by a grant from Korea Health Industry Development Institute (grant number HI17C0178).”

The correction will appear in the online version of the paper on the JMIR website on December 20, 2018, together with the publication of this correction notice. Because this was made after submission to PubMed, PubMed Central, and other full-text repositories, the corrected article also has been resubmitted to those repositories.

