# Supplementary material for: A Cardiopulmonary Monitoring System for Patient Transport Within Hospitals Using Mobile Internet of Things Technology: Observational Validation Study
Source: JMIR Mhealth Uhealth. 2018 Nov 14;6(11):e12048. doi: 10.2196/12048 (PMC6262206; doi:10.2196/12048)
Supplement: Multimedia Appendix 1 [file mhealth_v6i11e12048_app1.pdf]

Supplemental table 1 Number of raw, matched and imputation records from two wearable devices for twenty-three patients

SCR No	Type of Network	Raw data		Matched data			imputation		
		**Number of records from NONIN**	**Number of records from Prince-100H**	**Start time ~ end time**	**Duration (second)**	**Number of records**	**Before Number of NA (%*)**	**After Number of NA (%*)**	**Ratio of remaining NA (%)**
P01	WIFI	722	2395	10:07:50 AM : 10:53:58 AM	2768	693	88	0	0.00%
P02	WIFI	1959	3796	11:19:11 AM : 1:26:15 PM	7624	1907	965	89	4.67%
P03	LTE	875	2795	8:51:00 AM : 9:46:56 AM	3356	840	145	107	12.74%
P04	LTE	472	1470	9:51:25 AM : 10:15:57 AM	1472	369	3	3	0.81%
P05	LTE	524	762	8:47:05 AM : 9:19:37 AM	1952	489	298	137	28.02%
P06	LTE	654	4104	8:20:35 AM : 9:01:51 AM	2476	620	10	7	1.13%
P07	LTE	544	1483	9:00:25 AM : 9:31:29 AM	1864	467	102	68	14.56%
P08	LTE	604	1906	9:48:13 AM : 10:25:41 AM	2248	563	87	54	9.59%
P09	LTE	241	3370	7:55:24 AM : 8:10:16 AM	892	224	0	0	0.00%
P10	LTE	402	1465	9:16:42 AM : 9:41:10 AM	1468	368	3	3	0.82%
P11	LTE	121	1006	9:01:31 AM : 9:07:47 AM	376	95	1	1	1.05%
P12	LTE	750	2648	9:16:21 AM : 10:02:37 AM	2776	695	33	25	3.60%
P13	LTE	1716	6633	10:26:57 AM : 12:18:45 PM	6708	1678	22	17	1.01%
P14	LTE	218	764	11:22:35 AM : 11:35:19 AM	764	192	0	0	0.00%
P15	LTE	320	795	8:53:26 AM : 9:09:38 AM	972	244	44	2	0.82%
P16	LTE	438	1333	2:37:01 PM : 2:59:21 PM	1340	336	3	3	0.89%
P17	LTE	206	627	10:21:40 AM : 10:34:04 AM	744	187	33	1	0.53%
P18	LTE	231	647	9:42:48 AM : 9:55:16 AM	748	189	29	3	1.59%
P19	LTE	733	2616	10:27:31 AM : 11:14:23 AM	2812	704	49	32	4.55%
P20	LTE	369	1366	3:45:17 PM : 4:08:09 PM	1372	344	0	0	0.00%
P21	LTE	651	1920	10:04:20 AM : 10:44:00 AM	2380	597	597	2	0.34%
P22	LTE	626	2360	1:43:57 PM : 2:23:25 PM	2368	593	2	2	0.34%
P23	LTE	785	3022	10:13:08 AM : 11:03:36 AM	3028	759	1	1	0.13%
Total (Mean ± SD)		14161 (615.6±440.8)	49283 (2142.7±1417.4)	52508 (2282.9±1761.6)		13153 (571.8±440.3)	2515 (109.3±229.2)	557 (24.2±39.4)	4.23% (3.7±6.6)

* The denominator is the number of records in the matched data, the numerator is the number of NA records
